# Exploring the Mahuang Fuzi Xixin Decoction’s mechanism for treating Alzheimer’s disease using molecular docking and network pharmacology

**DOI:** 10.3389/fnagi.2025.1688316

**Published:** 2025-12-10

**Authors:** Che Chen, Qianfeng Shao, Sheng Zhou

**Affiliations:** 1 School of Traditional Chinese Medicine, Ningxia Medical University, Yinchuan, China; 2 Shenzhen Bao’an Chinese Medicine Hospital, Guangzhou University of Chinese Medicine, Shenzhen, China; 3 Qingdao Ruyi Software Co., Ltd., Medical Data Analysis Center, Qingdao, China

**Keywords:** Alzheimer’s disease, network pharmacology, molecular docking, Mahuang Fuzi Xixin Decoction, MMP9

## Abstract

**Objective:**

Explore the potential mechanism of Mahuang Fuzi Xixin Decoction (MFXD) in the treatment of Alzheimer’s disease (AD) using network pharmacology, molecular docking approaches, and test its efficacy by *in vitro* experiments.

**Methods:**

Active components of MFXD were screened from TCMSP, BATMAN-TCM, and TCMID, with corresponding targets obtained from SwissTargetPrediction and TCMSP. AD-related differential genes were retrieved from GEO. Intersection targets were identified via Venn diagrams, followed by GO/KEGG enrichment analyses, PPI network construction, and molecular docking. *In vitro* validation experiments were carried out using PC12 cells induced by Aβ_25–35_ to simulate the pathological state of AD. For the detection of cell viability, the CCK-8 assay was employed to evaluate the protective effect of MFXD and its active components on damaged PC12 cells. Western blot analysis was used to determine the protein expression levels of key molecules involved in AD-related signaling pathways, including phosphorylated p-NF-κB p65, NF-κB p65, p-GSK-3β, GSK-3β, MMP-9, p-Tau, and Tau. Additionally, the ELISA was utilized to measure the secretion level of TNF-α in the supernatant of Aβ_25–35_-induced PC12 cells, so as to assess the anti-inflammatory effect of MFXD.

**Results:**

Thirty-seven active components and 230 targets of MFXD were identified, along with 4913 AD-related differentially expressed genes from GEO dataset GSE122063, yielding 47 intersection targets. GO annotation enriched these targets in processes like reactive oxygen species metabolism, components like extracellular matrix, and functions like neurotransmitter binding; several pathways were enriched in the KEGG analysis, such as TNF signaling pathway, calcium signaling pathway, and NF-κB signaling pathway. The intersection target PPI network identified MMP9, EGFR, FOS as core targets. Molecular docking results indicated that quercetin binds to the three core targets (MMP9, EGFR, FOS), while luteolin binds preferentially to EGFR and MMP9. *In vitro*, Aβ_25–35_-induced PC12 cells treated with quercetin/luteolin had concentration-dependent viability increases (all *P* < 0.001); 15% MFXD-containing serum restored viability to ≥ 95% (P < 0.001 vs. AD model, comparable to DHCL). Western blot showed AD model had elevated p-NF-κB p65/NF-κB p65, MMP9/β-actin, p-Tau/Tau and reduced p-GSK-3β/GSK-3β (all *P* < 0.05); MFXD reversed these (all *P* < 0.05), while DHCL only inhibited p-NF-κB p65/NF-κB p65. ELISA showed MFXD and DHCL both reduced AD model’s TNF-α (all *P* < 0.001).

**Conclusion:**

MFXD potentially exerts anti-AD effects through a multi-component, multi-target, multi-pathway approach. Its key active components (quercetin, luteolin) may act by modulating the core target MMP9. Also, MFXD can simultaneously regulate several pathways, such as the TNF signaling pathway, Calcium signaling pathway, and NF-κB signaling pathway, and target Tau protein-related pathology by restoring the phosphorylation level of GSK-3β to suppress abnormal hyperphosphorylation of Tau, and thereby alleviating pathological damage in AD.

## 1 Introduction

Alzheimer’s disease (AD) is a neurodegenerative disorder primarily characterized by progressive cognitive impairment and behavioral decline. It is more prevalent in individuals aged 65 and older, with its incidence continuing to rise. As population aging intensifies, AD has emerged as a pressing global public health challenge (Hao and Chen, 2025). The pathogenesis of AD remains unsettled; its major pathological hallmarks include cerebral neuroinflammation, mitochondrial dysfunction, neurofibrillary tangles arising from hyperphosphorylated Tau (Protein Tau), and senile plaques formed by amyloid-β (Aβ) deposition (Meng et al., 2019; Pascoal et al., 2021; Ashleigh et al., 2023). At present, clinical drugs can merely ameliorate symptoms, decelerate progression, or preserve residual brain function (Livingston et al., 2020; Yiannopoulou and Papageorgiou, 2020; Khan et al., 2025). Moreover, most conventional chemical medicines are single-target agents accompanied by pronounced adverse effects and unsatisfactory long-term efficacy (Sharma, 2019). Traditional Chinese medicine (TCM), characterized by multi-level, multi-pathway, multi-target actions, can synergize with Western drugs to compensate for their shortcomings and has therefore become a promising strategy against AD. In TCM, AD falls under the categories of “dementia.” The pathogenesis is ascribed to kidney deficiency exhausting yang, so the brain loses its nourishment and the “Shen” (mental clarity) becomes dull, resulting in dementia and poor memory. *Inner Classics by Yellow Emperor* (the foundational work of TCM theoretical system, also called *Huangdi Neijing* in Chinese) states, “the brain is the residence of Shen”; Shen, as the commander of mental activities, governs consciousness, thinking, and memory—core functions impaired in AD—and deficient yang may aggravate cognitive decline. This book also states, “when yang-qi is abundant and ascending, it can refine itself to nourish the Shen…” Therefore, treatment should therefore focus on strengthening Yang, replenishing kidney essence, and restoring mental clarity.

Mahuang Fuzi Xixin Decoction (MFXD; Ephedra, Aconiti and Asarum Decoction), a well-known TCM formula, was first recorded from the monograph named *Treatise on Typhoid* and *Miscellaneous Diseases* during Han Dynasty about 2000 years ago in great China. This Decoction pairs Ephedra, Aconite and Asarum in a 2:3:1 ratio; in which the stems of Ephedra are removed and Aconite is pre-processed (soaked thoroughly in bittern solution, rinsed to remove partial aconitine, steamed until no white core, sliced and dried), and the three ingredients are decocted together to yield a warming, yang-lifting formula traditionally used for “dual Taiyang–Shaoyin” afflictions. Each herb plays a distinct role in line with TCM compatibility: Ephedra, as the sovereign herb, disperses exterior cold, induces diaphoresis to relieve superficies syndrome, and dredges yang circulation to facilitate the nourishment of the brain orifices; Aconite warms kidney yang and dispels internal cold as the minister herb; Asarum, aromatic in nature and adept at promoting circulation, can communicate between internal and peripheral organs, functioning as the adjunct herb to enhance the formula’s efficacy. Some clinical studies have found that MFXD can elevate treatments effect to AD, and the herbs in the formula have been reported to contain neuroprotective active components and exert therapeutic effects on neurodegenerative diseases (Zhou et al., 2021; Wei and Gao, 2019; Yang et al., 2018).

It is reported that Ephedra extracts reduced blood–brain barrier disruption and cerebral edema in rats after subarachnoid hemorrhage (Zuo et al., 2015). Pharmacological studies show that β-asarone, the main active compound in Asarum, improves learning and memory in APP/PS1 mice and increases the expression of SYP and GluR1, exerting neuroprotective effects against AD (Denny Joseph and Muralidhara, 2013). Wang found in an in-vitro study that aconite at 0.4–100 mg/mL has potential therapeutic and preventive effects in an AD cell model (Wang et al., 2021) (e.g., β-asarone in Asarum enhances learning and memory in APP/PS1 mice; Aconite exerts protective effects on AD cell models). However, the active constituents of MFXD and their molecular mechanisms protecting against aging and AD remain to be further elucidated. Although MFXD has shown certain therapeutic potential for AD, and the herbs in this formula (e.g., Ephedra, Aconite, Asarum) have been confirmed to improve brain neuronal activity in AD treatment, the therapeutic quantification and evaluation of MFXD as a whole in treating AD still lacks in-depth research. Network pharmacology serves as a cost-effective alternative to traditional drug discovery, particularly in elucidating the complex relationships among the multiple components and targets of TCMs. Leveraging this approach, high-throughput bioinformatics enables the construction of a “drug–compound–target–disease” network model (Noor et al., 2022; Tang and Aittokallio, 2014).

Thus, this study firstly employs network pharmacology and molecular docking to screen MFXD’s active components, predict their corresponding targets, identify intersection targets with AD, and further pinpoint the potential core targets of MFXD in AD treatment. To further address the research gap of MFXD’s anti-AD mechanism, this study then verifies the binding affinity between core components and key targets via molecular docking. Finally, *in vitro* experiments are conducted in Aβ_25–35_-induced PC12 cells to evaluate MFXD’s effects on cell viability (via CCK-8), the expression of core proteins (p-NF-κB p65, NF-κB p65, p-GSK-3β, GSK-3β, MMP-9, p-Tau, and total Tau) via Western blot, and the secretion of inflammatory factor TNF-α (via ELISA), thereby providing quantitative evidence for MFXD’s therapeutic role in AD and revealing its underlying mechanism.

## 2 Materials and methods

### 2.1 The collection of active components and component-target network construction

Through the TCMID database,^1^ TCMSP database,^2^ and BATMAN-TCM database (Bioinformatics Analysis Tool for Molecular mechANism of Traditional Chinese Medicine), the medicinal materials of MFXD, namely Aconite, Ephedra, and Asarum, were searched and their corresponding chemical components were obtained. With the screening criteria of oral bioavailability (OB) ≥ 30% and drug-likeness (DL) ≥ 0.18, the main active components were selected (Xu et al., 2012). The targets corresponding to the above active components were collected through the Swiss Target Prediction database^3^ and the TCMSP database. The targets were searched in the UniProt database^4^ with “Homo sapiens” as the species screening condition to obtain the gene symbols (Gene symbol) of the targets related to MFXD. Finally, the network diagram of the active components and drug targets of MFXD was drawn using Cytoscape_v3.8.2 software.

### 2.2 Screening AD-related targets

Through the Gene Expression Omnibus database (GEO), a gene dataset was retrieved by searching with the keyword “Alzheimer’s disease.” Differential gene analysis was performed between the AD group and the normal group using the criteria of |log (FC)| > 0.5 and adjusted *P* < 0.05 to screen for differentially expressed genes (DEGs). This process identified targets related to Alzheimer’s disease.

### 2.3 Screening of interacting targets

The differentially expressed genes (DEGs) obtained for AD were used as targets. The intersection of these AD targets and the drug targets was then determined. The resulting intersection targets were considered as candidate drug-disease targets. Using Perl (a scripting language widely used in bioinformatics for data processing), the active components associated with these intersection targets were extracted. These data were then formatted into an input file for constructing an active component-target network.

### 2.4 Disease-related active ingredient target enrichment analysis

To explore the functional impact of candidate drugs on disease targets, enrichment analyses were conducted using Gene Ontology (GO) and the Kyoto Encyclopedia of Genes and Genomes (KEGG). Detailed functional enrichment of these targets was further performed with the DAVID database.^5^ The top 10 significant terms in each GO category—Biological Process (BP), Cellular Component (CC), and Molecular Function (MF)—and 10 KEGG pathways related to AD were examined.

### 2.5 AD pathway–target network construction

To systematically investigate the interactions between pathways and targets, an interaction network file was generated using Perl. The network was visualized with Cytoscape 3.9.1, and topological analysis of the targets was performed with the CytoNCA plugin to calculate each target’s degree centrality.

### 2.6 PPI network analysis of MFXD–disease targets

The STRING database^6^ was used to construct a core protein–protein interaction (PPI) network. When building the network, a confidence cutoff of 0.4 was set, the option to hide isolated nodes was selected to exclude targets with no interaction links. To further identify the key targets among the MFXD–disease targets, Cytoscape 3.9.1 was employed to build the PPI network and perform topological analyses. In the resulting network, “nodes” represent the shared targets and “edges” depict the interactions between them.

### 2.7 Molecular docking

Molecular docking was performed to validate the binding affinities of the key active ingredients quercetin and luteolin with the core targets epidermal growth factor receptor (EGFR), proto-oncogene protein (FOS), and matrix metalloproteinase-9 (MMP9). The 2-D SDF structures of the main active ingredients were downloaded from the PubChem platform,^7^ and their 3-D conformations were generated in ChemBio3D Ultra 14.0. The official UniProt website^8^ was queried with the core target gene names to retrieve the corresponding protein IDs, after which the protein structures of the key targets were obtained from the PDB database.^9^ Water molecules and small-molecule ligands were removed using PyMOL; receptor binding pockets were defined and their parameters adjusted with AutoDockTools-1.5.6. Docking was carried out with AutoDock Vina, running 20 independent trials for each complex and selecting the pose with the lowest binding free energy as the optimal result. Discovery Studio 2016 Client was employed to calculate inter-residue interaction forces within the docking site and to export both 3-D and 2-D diagrams of the interaction regions.

### 2.8 *In vitro* cellular assay validation

Based on the molecular docking results in section 2.7, the binding energy between MMP9 and luteolin is the lowest, indicating the highest probability of actual binding; quercetin also shows favorable binding to MMP9. Therefore, these two representative core components will be subjected to *in vitro* experimental validation to evaluate their potential therapeutic effects on AD.

#### 2.8.1 Materials

Dulbecco’s Modified Eagle’s Medium (DMEM; Gibco, Cat. No. 30022.01, Shanghai, China) and fetal bovine serum (FBS; Gibco, Cat. No. FB25015, Shanghai, China) were used for cell culture. PC12 cells were obtained from the Cell Bank of the Chinese Academy of Sciences (Shanghai, China). The Aβ_25–35_ peptide (Cat. No. A104154) and donepezil hydrochloride (DHCL, Cat. No. D108180) were purchased from Aladdin Biochemical Technology Co., Ltd. (Shanghai, China). The anti-MMP-9 antibody (Cat. No. ab228402) was obtained from Abcam (Cambridge, United Kingdom). The Cell Counting Kit-8 (CCK-8; Lot No. WLA074) was purchased from Wanleibio Co., Ltd. (Shenyang, China). Streptomycin and penicillin were obtained from Invitrogen (Carlsbad, CA, United States). The TNF-αELISA Kit (Cat. No. E-MSEL-M0002) was purchased from Elabscience (Wuhan, China). Primary antibodies against p-NF-κB p65 (Cat. No. 3033), NF-κB p65 (Cat. No. 3034), p-GSK-3β (Cat. No. bs-0028R), GSK-3β (Cat. No. bs-0027R), p-Tau (Cat. No. bs-5571R), and Tau (Cat. No. bs-0056R) were obtained from Beijing Boosen Biological Technology Co., Ltd. (Beijing, China)

#### 2.8.2 Preparation of MFXD-containing serum

Male Wistar rats (SPF, 6 weeks old, 20 ± 2 g; *n* = 20; Vital River, Beijing, China) were used. Ephedra sinica (Mahuang), Aconitum carmichaeli Debx (processed lateral roots, Fuzi), and Asarum heterotropoides var. mandshuricum (Xixin) were mixed in a ratio of 2:3:1. Mahuang was soaked in 15 volumes of water for 30 min, decocted for 20 min to remove foam, then combined with the other herbs and co-decocted for 90 min. The filtrate was collected and concentrated to 1.8 g/mL of crude drug.

For serum preparation, rats were fasted for 12 h and then administered the MFXD extract intragastrically at 18 g/kg. Control rats received the same volume of saline. Blood was collected from the abdominal aorta 30 min after administration, kept at room temperature for 2 h, and centrifuged at 3,000 rpm for 10 min. The serum was heat-inactivated (56°C 30 min), filtered through a 0.22 μm membrane, and stored at –20°C (Tang et al., 2015).

#### 2.8.3 Cell culture and grouping

After recovery, PC12 cells were maintained at 37 °C in a 5% CO2 incubator using DMEM supplemented with 10 % fetal bovine serum and 1% penicillin/streptomycin. Based on the literature (Zhen, 2021; Luo et al., 2019), the following groups were established:

(1) Normal control: untreated cells; (2) AD model: cells cultured for 4 days in serum-free medium containing 250 μM Aβ*25*–*35* (Mizoguchi et al., 2009; Changhong et al., 2021); (3) Quercetin: after the 4-day Aβ*25*–*35* exposure, cells were treated with 20, 40, or 60 μM quercetin for 24 h; (4) Luteolin : after the 4-day Aβ*25*–*35* exposure, cells were treated with 50, 70, or 90 μM luteolin for 24 h; (5) after the 4-day Aβ*25*–*35* exposure, cells were treated with MFXD-containing serum; (6) DHCL: after the 4-day Aβ*25*–*35* exposure, cells were treated with 50 μM donepezil hydrochloride for 24 h (Han et al., 2022).

#### 2.8.4 Effects of CCK-8 cell viability monitoring

PC12 cells were seeded at 1 × 104 cells/well into two sets of 96-well plates and incubated at 37 °C for 12 h. After exposure to 250 μM Aβ_25–35_ for 4 days to establish the AD model, the cells were treated with quercetin (20, 40, or 60 μM), luteolin (50, 70, or 90 μM), or MFXD-containing serum (5, 10, 15, or 20%). Quercetin and luteolin treatments were performed for 24 h, whereas MFXD-containing serum was administered for 4, 8, 12, 24, or 48 h. Cell viability was determined using the Cell Counting Kit-8 (CCK-8, Dojindo, Japan) according to the manufacturer’s instructions. Briefly, 10 μL of CCK-8 solution was added to each well and incubated for 2 h at 37°C, and the absorbance was measured at 450 nm using a microplate reader.

#### 2.8.5 Western blot analysis

Employ high-efficiency RIPA lysis buffer (Solarbio, R0010). Immediately prior to use, mix the reagents at a ratio of 1 mL RIPA buffer: 10 μL protease inhibitor (PMSF) to prepare the cell lysis working solution. After harvesting cells, add the working solution, centrifuge, and transfer the supernatant to boiling water for 10 min to obtain total cellular proteins. Protein samples are resolved by SDS-polyacrylamide gel electrophoresis and transferred onto polyvinylidene fluoride (PVDF) membranes. The membranes are incubated overnight at 4 °C with the primary antibody against MMP9 (1:1,000), phospho-NF-κB p65 (CST3033, 1:1000), NF-κB p65 (CST3034, 1:1,000), phospho-GSK-3β(CST9323, 1:1,000); GSK-3β(CST9315, 1:1,000); phospho-Tau (CST71429, 1:1,000); phospho-Tau (CST #71429, 1:1,000); total Tau (CST #4019, 1:1,000); and β-actin (Abcam ab8227, 1:1,000). After TBST washes, membranes were incubated with HRP-conjugated goat anti-rabbit or goat anti-mouse IgG (1:2,000; Wuhan Boster Biological Technology Co., Ltd.) for 1 h at room temperature. Bands were visualized by enhanced chemiluminescence (ECL) and quantified using the Lane ID gel analysis system.

#### 2.8.6 ELISA analysis

Concentrations of TNF-αin PC12 cell supernatants were quantified using ELISA kits (TNF-α: MM-0132M1, Enzyme-linked Biotechnology, Shanghai, China) according to the manufacturer’s instructions.

#### 2.8.7 Statistical analysis

All data were expressed as mean ± SD. Normality and homogeneity of variances were tested using the Shapiro–Wilk and Levene’s tests, respectively. Differences between groups were analyzed by Student’s *t*-test or one-way ANOVA; nonparametric rank-sum tests were applied when assumptions were not met. Statistical analysis was performed using GraphPad Prism 9.0 (GraphPad Software, United States), and *P* < 0.05 was considered statistically significant.

## 3 Results

### 3.1 Active components collection and component-target network construction

**FIGURE 1:**
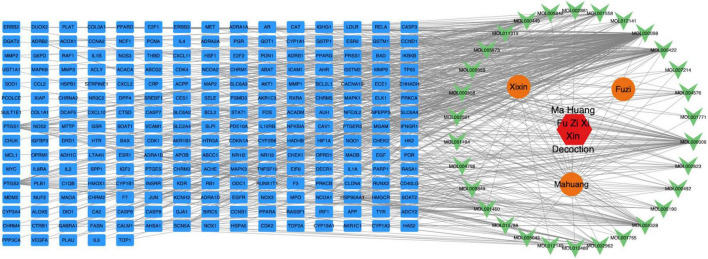
Network of active constituents and drug targets of Mahuang Fuzi Xixin Decoction.

Database screening results revealed a total of 37 primary active constituents in MFXD, including 23 from Ephedrae, 8 from Asarum, and 6 from Aconiti. Subsequent mapping of these active components yielded 230 corresponding targets. All targets were imported into UniProt to retrieve their official gene symbols. The dataset was then loaded into Cytoscape 3.9.1 to construct the herbal compound–target network, as illustrated in Figure 1.

### 3.2 Identification of AD differentially expressed genes

**FIGURE 2:**
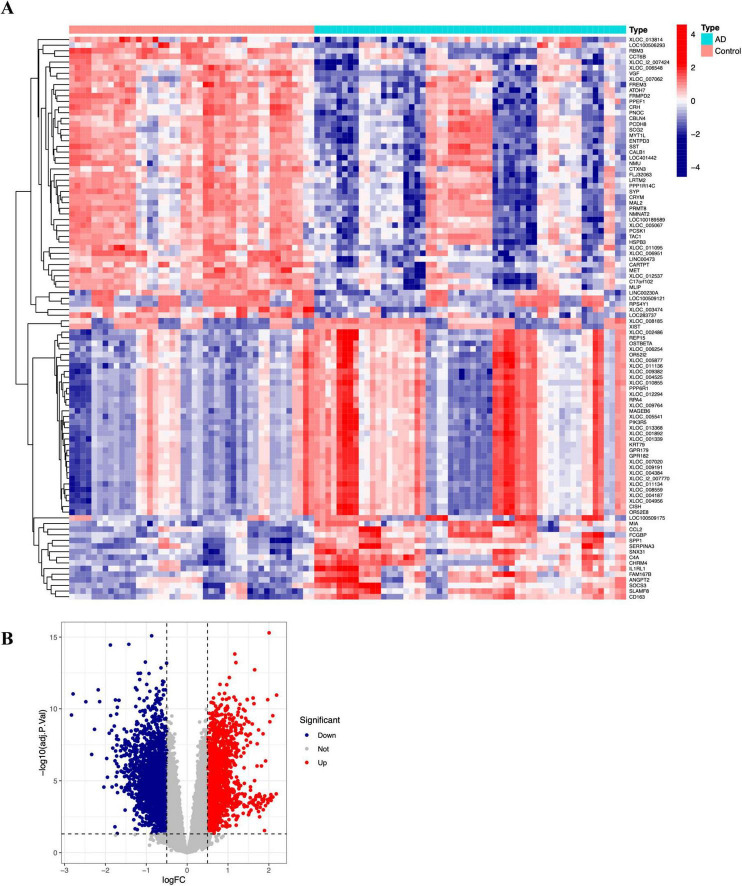
Differential gene analysis of the GSE122063 dataset. (A) Volcano plot of the top 100 differentially expressed genes. (B) Heatmap of the top 100 differentially expressed genes.

The GSE122063 transcriptomic microarray dataset for Alzheimer’s disease was obtained from the GEO database; it comprises 44 normal controls and 56 AD samples derived from brain tissue. A total of 2,599 down-regulated genes and 2,314 up-regulated genes were identified. The top 100 differentially expressed genes were visualized as a heatmap using the heatmap package, and a volcano plot was generated with the ggplot2 package, as shown in Figures 2A,B), respectively.

### 3.3 Identification of AD active ingredient targets

**FIGURE 3:**
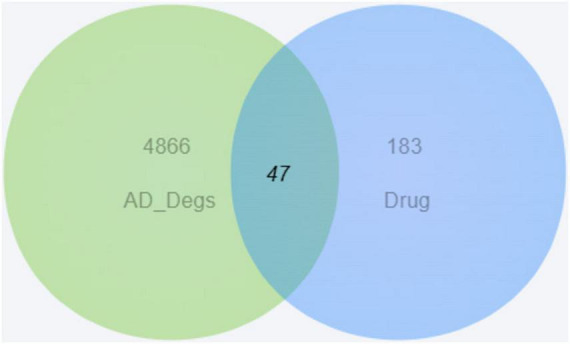
Venn diagram of common targets between Mahuang-Fuzi-Xixin Decoction and Alzheimer’s disease (AD).

**FIGURE 4:**
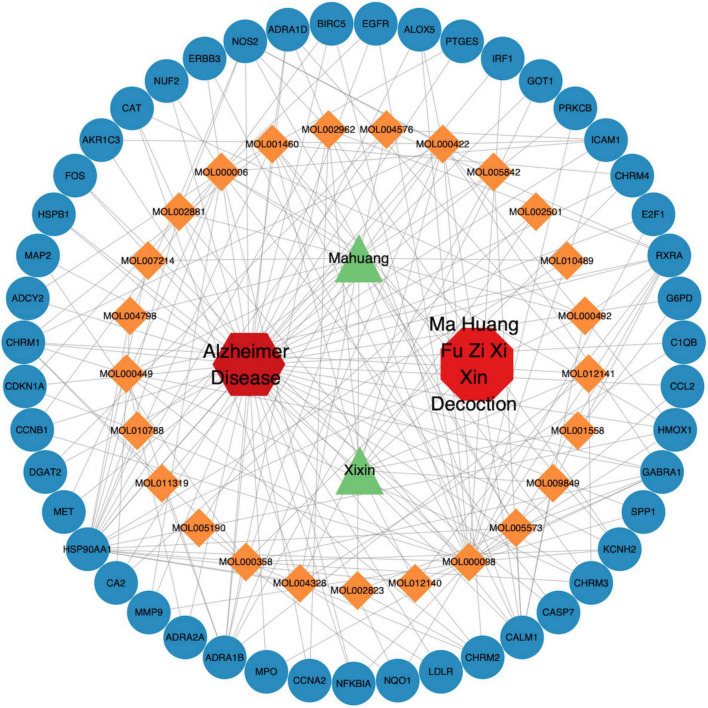
Integrated drug-disease-compound-target network of Mahuang-Fuzi-Xixin Decoction and Alzheimer’s disease.

Using differentially expressed genes as AD disease targets and intersecting them with the component targets of MFXD yielded 47 overlapping targets (Figure 3). These were taken as the drug-active-ingredient disease targets; the drug–disease–active-ingredient–target network file was prepared with Perl and visualized with Cytoscape 3.9.1. Among them, 25 active ingredients and 2 Chinese medicinal participated in the network construction, as shown in Figure 4.

### 3.4 GO/KEGG Enrichment analysis

**FIGURE 5:**
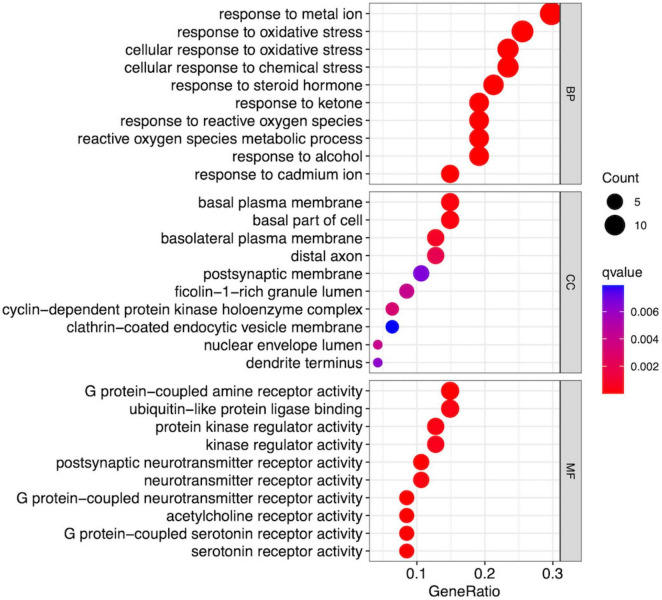
Bubble plot of top 10 GO pathways.

To determine the functions of the candidate targets, GO and KEGG enrichment analyses were performed. The cluster Profiler and org.Hs.eg.db packages were employed to conduct enrichment analysis on the disease-active-ingredient targets, and the top 10 results were visualized. Significant enrichment results were screened according to the criteria of adjusted *P* > 0.05 and count > 1. GO annotation revealed 706 significant results in the Biological Process (BP) category, 50 in the Cellular Component (CC) category, and 55 in the Molecular Function (MF) category; the top 10 of these GO results were visualized as circle plots, as shown in Figure 5.

**FIGURE 6:**
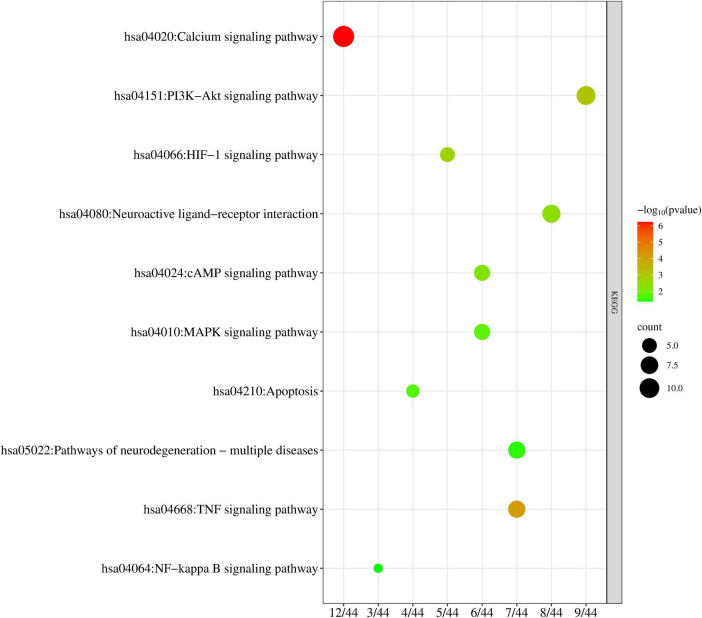
Bubble plot of 10 KEGG pathways associated with Alzheimer’s disease (AD).

BP top 10 results were significantly enriched in entries related to reactive oxygen species metabolism, metal ion metabolism, and compound metabolism. CC top 10 results were enriched considerably in entries related to extracellular matrix structure, synaptic structure, cell cycle protease complex, etc. MF top 10 results were significantly enriched on entries about neurotransmitter receptors and proteases. The KEGG enrichment results can more intuitively explain the relationship between targets and pathways, with 116 significant KEGG pathways identified; 10 KEGG signaling pathways related to AD are shown in Table 1 and Figure 6.

**TABLE 1:** Ten KEGG pathways associated with the Alzheimer’s disease (AD) field.

ID	Description	P.adjust	Gene ID	Count
Hsa04020	Calcium signaling pathway	5.8645E-07	MET/CHRM1/CHRM3/NOS2/ ADRA1B/EGFR/ADRA1D/ADCY2/ ERBB3/PRKCB/CALM1/CHRM2	12
Hsa04668	TNF signaling pathway	6.4202E-05	CCL2/NFKBIA/CASP7/IRF1/ICAM1/FOS/MMP9	7
Hsa04151	PI3K-Akt signaling pathway	0.00084964	SPP1/HSP90AA1/MET/CHRM1/RXRA/EGFR	9
			/ERBB3/CDKN1A/CHRM2	
hsa04066	HIF-1 signaling pathway	0.00189746	HMOX1/NOS2/EGFR/PRKCB/CDKN1A	5
Hsa04080	Neuroactive ligand-receptor interaction	0.00394036	CHRM4/CHRM1/CHRM3/ADRA1B /ADRA1D/	8
			ADRA2A/GABRA1/CHRM2	
Hsa04024	cAMP signaling pathway	0.00591266	CHRM1/NFKBIA/ADCY2/FOS/CALM1/CHRM2	6
Hsa04010	MAPK signaling pathway	0.01655475	HSPB1/MET/EGFR/ERBB3/PRKCB/FOS	6
Hsa04210	Apoptosis	0.01963358	NFKBIA/CASP7/BIRC5/FOS	4
Hsa05022	Pathways of neurodegeneration-multiple diseases	0.0330773	CHRM1/CHRM3/NOS2/CASP7/CAT/PRKCB/CALM1	7
Hsa04064	NF-kappa B signaling pathway	0.04123143	NFKBIA/PRKCB/ICAM1	3

### 3.5 AD pathway–target network construction

**FIGURE 7:**
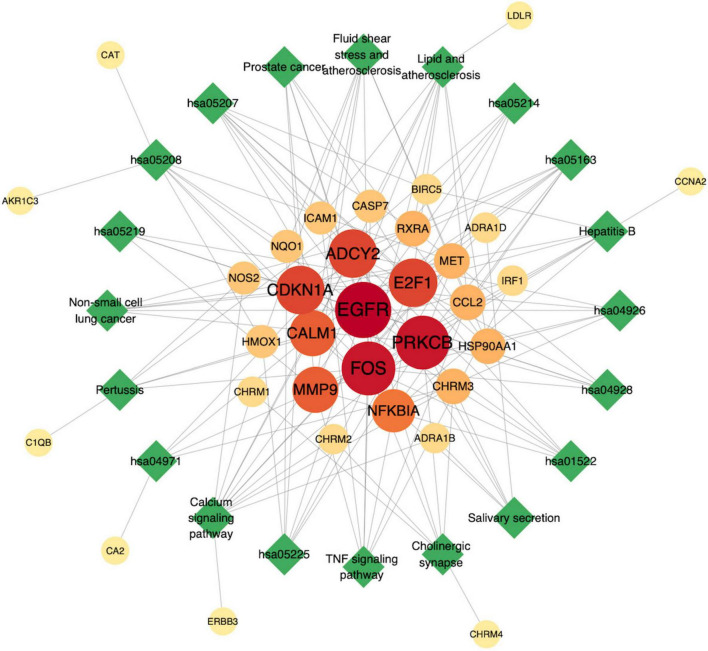
AD pathway–target network.

The AD pathway–target network analysis results are shown in Figure 7. Node size and color were visualized according to degree values; darker and larger nodes indicate greater importance of the target within the pathway–target network. As shown in the figure, the top three targets were EGFR, PRKCB, and FOS (Figure 7).

### 3.6 Screening of PPI core targets

**FIGURE 8:**
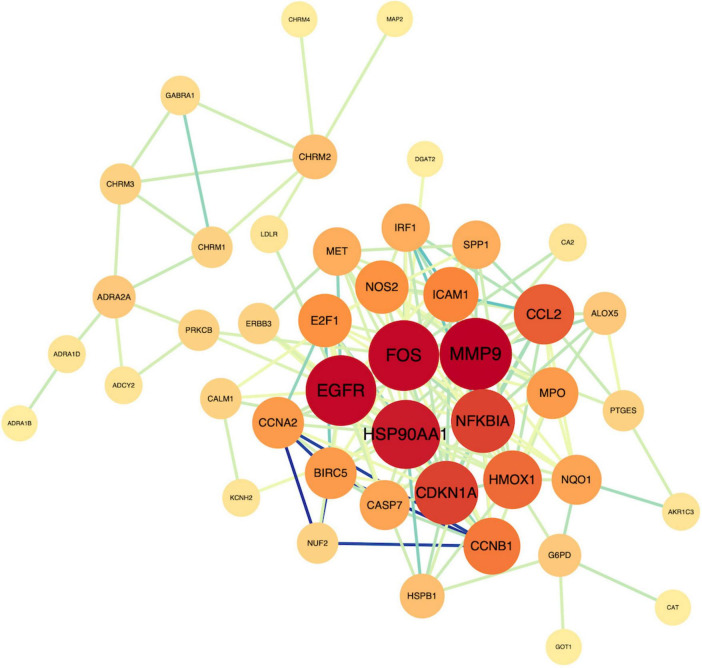
Intersection target network diagram. Nodes represent the shared targets, and Edges depict the interactions between them.

**FIGURE 9:**
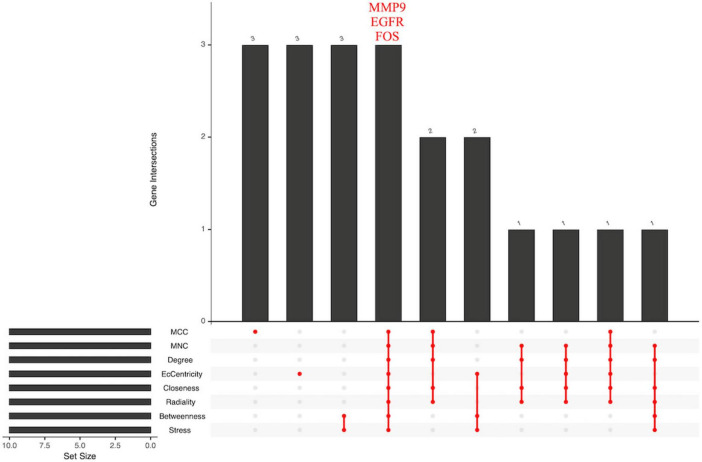
Core target UPSET plot.

The STRING database was employed to construct a core-target protein–protein interaction (PPI) network, which was visualized and subjected to topological analysis using Cytoscape 3.9.1. The primary network comprised 45 nodes and 171 edges. The cytoNCA plug-in was applied to calculate the node degree values, with larger nodes indicating higher degrees. Subsequently, the cytoHubba plug-in performed topological analyses, yielding the topological parameters listed in Table 2: MCC, MNC, Degree, Eccentricity, Closeness, Radiality, Betweenness, and Stress. The top 10 genes ranked for each parameter were intersected, ultimately designating MMP9, EGFR, and FOS as the core targets. These three targets are not only drug targets of MFXD but also pivotal components of AD-related signaling pathways, exerting critical roles in AD therapy, as illustrated in Figures 8, 9.

**TABLE 2:** Topological parameters of the top 10 core targets.

Node_name	MCC	MNC	Degree	EcCentricity	Closeness	Radiality	Betweenness	Stress
MMP9	128,480	22	22	0.200	30.367	6.227	164.008	732
EGFR	101,945	20	21	0.250	30.750	6.409	629.839	1,726
FOS	83,093	20	21	0.250	30.500	6.341	365.800	1,024
HSP90AA1	106,818	20	20	0.200	29.367	6.182	159.012	668
CDKN1A	121,086	17	17	0.200	27.700	6.091	41.772	280
NFKBIA	46,968	17	17	0.200	27.867	6.114	57.766	376
CCNB1	90,888	13	13	0.200	25.367	5.932	20.572	134
E2F1	90,744	11	11	0.200	24.367	5.886	4.118	24
BIRC5	80,664	10	10	0.200	23.867	5.864	6.959	58
CCNA2	80,664	10	10	0.200	23.867	5.864	6.959	58

### 3.7 Molecular docking of core targets with active pharmaceutical ingredients

**FIGURE 10:**
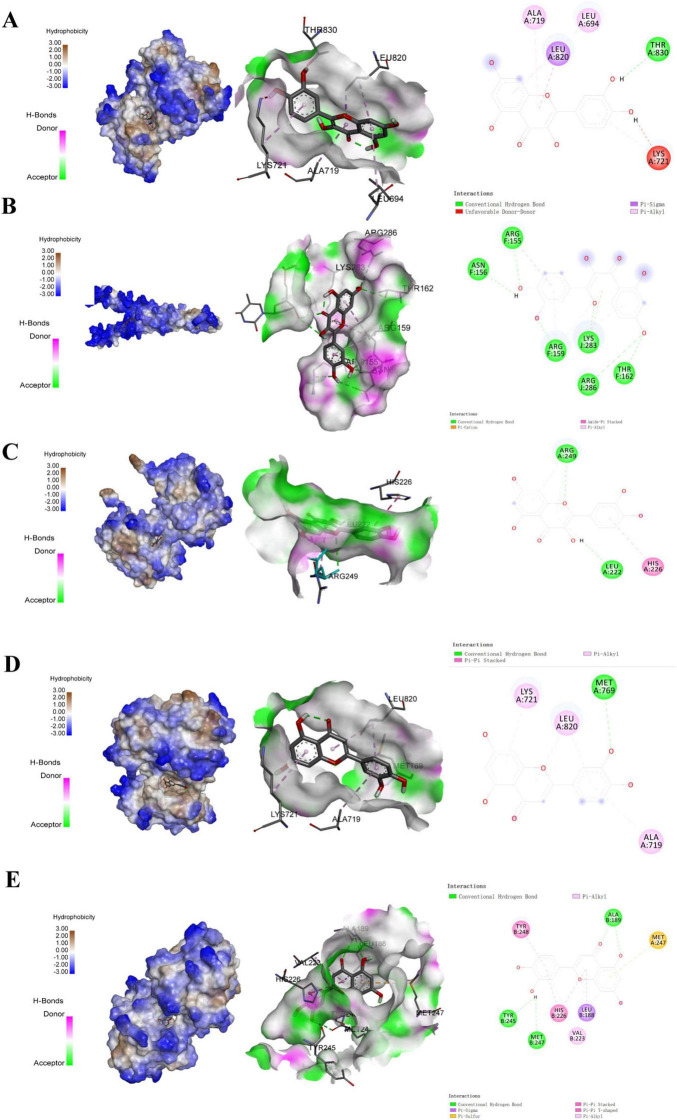
Schematic diagrams of molecular docking between the core active ingredients and their respective targets. (A) Molecular docking of quercetin-EGFR. (B) Molecular docking of quercetin-FOS. (C) Molecular docking of quercetin-MMP9. (D) Molecular docking of Luteolin-EGFR. (E) Molecular docking of Luteolin-MMP9.

Molecular docking between the core targets and their active ingredients revealed that quercetin and luteolin are the key bioactive compounds. Quercetin exhibits favorable binding affinities to all three top-ranked targets (EGFR, FOS, and MMP9). In contrast, luteolin preferentially binds to EGFR and MMP9 with superior affinities compared with other targets, as shown in Figures 10A–E. The optimal models were selected based on the lowest binding energies and subsequently exported as three-dimensional representations. The binding energies and the intermolecular forces within the docking pockets are summarized in Table 3. A binding energy below -5.0 kcal mol^–1^ indicates that interaction between the target and the active ingredient is energetically favorable; lower energies denote higher binding probabilities. An additional metric for evaluating docking reliability is the number of hydrogen bonds formed in the binding site, and no less than 2 hydrogen bonds are considered indicative of effective binding, with higher counts implying stronger interactions. According to these criteria, all optimal models demonstrated potential binding. Among them, the MMP9–luteolin complex exhibited the lowest binding energy, while the quercetin–FOS complex formed the highest number of hydrogen bonds.

**TABLE 3:** Active-site pocket coordinates and docking results for core targets.

Ligand	Receptor	center_x	center_y	center_z	size_x	size_y	size_z	kcal/mol	Total interactions
Quercetin	EGFR	56	62	58	23.552	9.728	58.840	-7.300	7
Quercetin	FOS	52	32	84	40.092	34.178	63.605	-6.000	11
Quercetin	MMP9	58	64	50	25.632	-1.214	19.024	-10.500	5
Luteolin	EGFR	56	62	58	23.552	9.728	58.840	-7.700	5
Luteolin	MMP9	58	64	50	25.632	-1.214	19.024	-11.000	13

### 3.8 *In vitro* experimental validation results

#### 3.8.1 Effects of core active components on cell viability

##### 3.8.1.1 Impact of quercetin on the viability of AD model cells

CCK-8 assay demonstrated that, compared with the normal control group, the viability of AD model cells was significantly reduced (*P* < 0.001). Relative to the AD model group, all quercetin-treated groups exhibited a concentration-dependent and statistically significant increase in cell viability (*P* < 0.001), as detailed in Table 4.

**TABLE 4:** Effect of quercetin on cell viability in Alzheimer’s disease model ($\bar{X}$ ± *s*).

Group	*n*	Cell viability
Normal group	8	100 ± 0.00%
AD model group	8	73 ± 3.00%^△△△^
Quercetin low-dose group	8	86 ± 1.00%***
Quercetin medium-dose group	8	93 ± 1.95%***
Quercetin high-dose group	8	99 ± 1.36%***

Compared with the normal group, ^△△△^ *P* < 0.001; compared with the AD model group, ***P < 0.001.

##### 3.8.1.2 Impact of luteolin on the viability of AD model cells

CCK-8 assay revealed that, relative to the normal group, the viability of AD model cells was markedly decreased (*P* < 0.001). In contrast, treatment with various concentrations of luteolin significantly elevated cell viability compared with the AD model group, with all differences achieving statistical significance (*P* < 0.001; see Table 5).

**TABLE 5:** Effect of luteolin on cell viability in Alzheimer’s disease model ($\bar{X}$ ± *s*).

Group	*n*	Cell viability
Normal group	8	100 ± 0.00%
AD model group	8	70 ± 1.14%^△△△^
Luteolin low-dose group	8	79 ± 0.77%***
Luteolin medium-dose group	8	85 ± 1.11%***
Luteolin high-dose group	8	95 ± 2.36%***

Compared with the normal group, ^△△△^ *P* < 0.001; compared with the AD model group, ***P < 0.001.

##### 3.8.1.3 Effects of MFXD on cell viability

**FIGURE 11:**
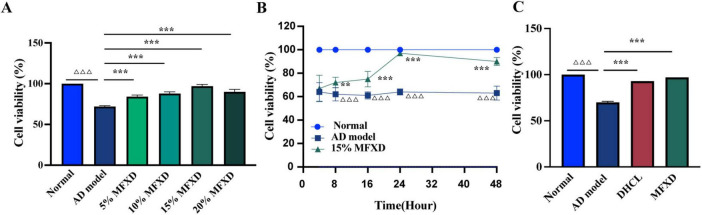
Effects of Mahuang Fuzi Xixin Decoction on the viability on cell viability in Alzheimer’s disease model. (A) Dose–response curve showing cell viability following 5, 10, 15, and 20% MFXD treatment for 24 h. (B) Time–course analysis of 15% MFXD intervention at 4, 8, 12, 24, and 48 h. (C) Comparative effects of 15% MFXD and donepezil hydrochloride (DHCL, 50 μM) on cell viability after 24 h. Cell viability was determined by CCK-8 assay and expressed as a percentage relative to the normal control group. Data are presented as mean ± SD (*n* = 3). ^△△△^ *P* < 0.001 vs. the Normal group; **P < 0.01, ***P < 0.001 vs. the AD model group.

To establish an experimental framework for mechanistic investigations of MFXD, we first defined its optimal concentration and exposure window. CCK-8 assays demonstrated that MFXD dose-dependently rescued the viability of Aβ-insulted SH-SY5Y cells; 15 % (v/v) MFXD was the most effective, restoring survival to ≥ 95% (Table 6 and Figure 11A). Time-course analysis showed that viability in the normal control group remained significantly higher than in the Aβ-damaged group at every interval (*P* < 0.001). Treatment with 15 % MFXD significantly reversed the deficit after 8 h (*P* < 0.01), with efficacy increasing at 16 h and 24 h (both *P* < 0.001), peaking at 24 h when survival again reached ≥ 95% (*P* < 0.001 vs. Aβ group; Table 7 and Figure 11B). Accordingly, 15 % MFXD for 24 h was adopted as the standard condition for subsequent experiments. As shown in Table 8, Figure 11C, the Aβ-exposed group exhibited markedly lower viability than the normal control (*P* < 0.001), confirming the above findings; compared with the Aβ group, both MFXD and donepezil hydrochloride (DHCL) significantly elevated cell survival (both *P* < 0.001), with the MFXD cohort slightly surpassing the DHCL group by restoring viability to ≥ 95 %.

**TABLE 6:** Effect of MFXD at different concentrations on cell viability in Alzheimer’s disease model ($\bar{X}$ ± *s*).

Group	*n*	Cell viability
Normal group	8	100 ± 0.00%
AD model group	8	71 ± 0.98%^△△△^
5% MFXD group	8	83 ± 2.10%***
10% MFXD group	8	87 ± 1.61%***
15% MFXD group	8	96 ± 0.94%***
20% MFXD group	8	89 ± 2.63%***

Compared with the normal group, ^△△△^ *P* < 0.001; compared with the AD model group, ***P < 0.001.

**TABLE 7:** Effect of 15%MFXD on cell viability in Alzheimer’s disease model at different time points ($\bar{X}$ ± *s*).

Group	N	4H Cell viability	8H Cell viability	16H Cell viability	24H Cell viability	48H Cell viability
Normal group	8	100 ± 0.00%	100 ± 0.00%	100 ± 0.00%	100 ± 0.00%	100 ± 0.00%
AD model group	8	64 ± 7.90%^△△△^	62 ± 5.75%^△△△^	61 ± 3.09%^△△△^	64 ± 2.62%^△△△^	63 ± 5.93%^△△△^
15% MFXD group	8	67 ± 11.29%	72 ± 4.50%**	75 ± 6.60%***	97 ± 1.05%***	90 ± 3.30%***

Compared with the normal group, ^△△△^ *P* < 0.001; compared with the AD model group, **P < 0.01, ***P < 0.001.

**TABLE 8:** Effect of MFXD at different concentrations on cell viability in Alzheimer’s disease model ($\bar{X}$ ± *s*).

Group	*n*	Cell viability
Normal group	8	100 ± 0.00%
AD model group	8	69 ± 0.01%^△△△^
DHCL group	8	93 ± 0.05%***
MFXD group	8	97 ± 0.05%***

Compared with the normal group, ^△△△^ *P* < 0.001; compared with the AD model group, ***P < 0.001.

#### 3.8.2 Western blot analysis

##### 3.8.2.1 Effects of core active components

**FIGURE 12:**
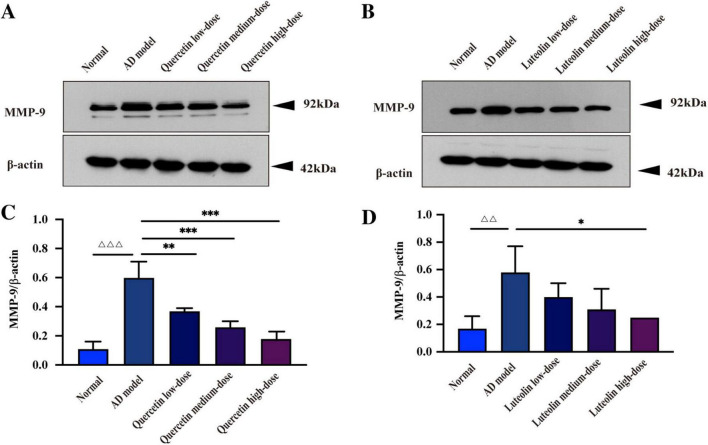
Effects of quercetin and luteolin on MMP9 protein expression. (A) Representative Western blot bands showing MMP-9 and β-actin expression after quercetin treatment. (B) Representative western blot bands showing MMP-9 and β-actin expression after luteolin treatment. (C,D) Quantitative analysis of MMP-9/β-actin ratios in quercetin-treated and luteolin-treated groups, respectively. Data are presented as mean ± SD (*n* = 3). ^△△^*P* < 0.01, ^△△△^*P* < 0.001 vs. Normal group; *P < 0.05, **P < 0.01, ***P < 0.001 vs. AD model group.

Western blot results following quercetin or luteolin treatment in AD model cells are shown in Figures 12A–D and Tables 9, 10. Compared with the normal group, MMP9 protein levels and the MMP9/β-actin ratio in the AD model group were markedly elevated (*P* < 0.001). Relative to the AD model group, quercetin at high (*P* < 0.001), medium (*P* < 0.001), and low (*P* < 0.01) doses significantly reduced both MMP9 protein expression and the MMP9/β-actin ratio. Similarly, in luteolin-treated groups, MMP9 levels and the MMP9/β-actin ratio were significantly increased in the AD model group versus the normal group (*P* < 0.01); compared with the AD model group, high-dose luteolin markedly down-regulated MMP9 protein expression and the MMP9/β-actin ratio (*P* < 0.05).

**TABLE 9:** Effect of quercetin on MMP9 protein expression in AD model cells ($\bar{X}$ ± *s*).

Group	*n*	MMP9/β -actin
Normal group	3	0.11 ± 0.05
AD model group	3	0.60 ± 0.11^△△△^
Quercetin low-dose group	3	0.37 ± 0.02**
Quercetin medium-dose group	3	0.26 ± 0.04***
Quercetin high-dose group	3	0.18 ± 0.06***

Compared with the normal group, ^△△△^ *P* < 0.001; compared with the AD model group, **P < 0.01, ***P < 0.001.

**TABLE 10:** Effect of Luteolin on MMP9 protein expression in AD model cells ($\bar{X}$ ± *s*).

Group	*n*	MMP9/β -actin
Normal group	3	0.17 ± 0.09
AD model group	3	0.58 ± 0.19^△△^
Luteolin low-dose group	3	0.40 ± 0.10
Luteolin medium-dose group	3	0.31 ± 0.15
Luteolin high-dose group	3	0.25 ± 0.17*

Compared with the normal group, ^△△^ *P* < 0.01; compared with the AD model group, *P < 0.05.

##### 3.8.2.2 Effects of MFXD

**FIGURE 13:**
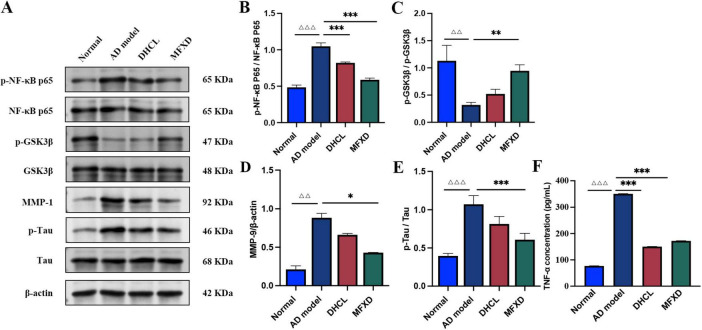
Effect of Mahuang Fuzi Xixin Decoction protein expression in AD model cells. (A) Representative Western blots showing expression of p-NF-κB p65, NF-κB p65, p-GSK-3β, GSK-3β, MMP-9, p-Tau, and total Tau, with β-actin as the internal control. (B–E) Quantitative analysis of the ratios p-NF-κB p65/NF-κB p65, p-GSK-3β/GSK-3β, MMP-9/β-actin, and p-Tau/Tau, respectively. (F) TNF-α concentrations in cell supernatants measured by ELISA. Data are expressed as mean ± SD (*n* = 3). ^△△^ *P* < 0.01, ^△△△^ *P* < 0.001 vs. the Normal group; *P < 0.05, **P < 0.01, ***P < 0.001 vs. the AD model group.

As shown in Table 11, Figures 13A–E, the AD model group exhibited significant elevations in the ratios of p-NF-κB p65/NF-κB p65, MMP-9/β-actin, and p-Tau/Tau, together with a marked reduction in p-GSK-3β/GSK-3β (vs. Normal group, *P* < 0.001, *P* < 0.01, *P* < 0.001, and *P* < 0.01, respectively), indicative of aggravated neuroinflammation and aberrant Tau hyper-phosphorylation. These alterations were substantially reversed following treatment with either MFXD or donepezil hydrochloride (DHCL). Relative to the AD model group, DHCL selectively down-regulated p-NF-κB p65/NF-κB p65 (*P* < 0.001), whereas MFXD concurrently reduced p-NF-κB p65/NF-κB p65, MMP-9/β-actin, and p-Tau/Tau (*P* < 0.001, *P* < 0.05, and *P* < 0.001, respectively) and restored p-GSK-3β/GSK-3β (*P* < 0.01). Notably, the recovery of GSK-3β phosphorylation and Tau dephosphorylation was more pronounced under MFXD than under DHCL, suggesting that the neuroprotective efficacy of MFXD is mediated, at least in part, through simultaneous suppression of the NF-κB/MMP-9 inflammatory axis and modulation of the GSK-3β/Tau signaling cascade.

**TABLE 11:** Effect of MFXD protein expression in AD model cells ($\bar{X}$ ± *s*).

Group	n	p-NF-κ B p65/NF-κ B p65	p-GSK3β /GSK3β	MMP-9/β -actin	p-Tau/Tau
Normal group	3	0.48 ± 0.03	1.13 ± 0.28	0.21 ± 0.05	0.40 ± 0.03
AD model group	3	1.05 ± 0.05^△△△^	0.32 ± 0.05^△△^	0.88 ± 0.06^△△^	1.07 ± 0.11^△△△^
DHCL group	3	0.82 ± 0.01***	0.52 ± 0.09	0.66 ± 0.02	0.81 ± 0.10
MFXD group	3	0.59 ± 0.02***	0.95 ± 0.11**	0.43 ± 0.00*	0.61 ± 0.08***

Compared with the normal group, ^△△^ *P* < 0.01, ^△△△^ *P* < 0.001; compared with the AD model group, *P < 0.05, **P < 0.01, ***P < 0.001.

##### 3.8.2.3 ELISA analysis results

As shown in Table 12, Figure 13F, the TNF-α level in the AD model group was markedly elevated compared with the normal group (*P* < 0.001), indicating a pronounced inflammatory response triggered by Aβ*25*–*35*. Following treatment with either MFXD or donepezil hydrochloride (DHCL), TNF-α secretion was significantly reduced relative to the AD model group (both *P* < 0.001).

**TABLE 12:** Effect of MFXD on TNF-α levels in AD model cells ($\bar{X}$ ± *s*).

Group	n	TNF-α (pg/mL)
Normal group	3	76.96 ± 0.56
AD model group	3	351.14 ± 0.80^△△△^
DHCL group	3	150.41 ± 0.66***
MFXD group	3	172.19 ± 0.70***

Compared with the normal group, ^△△△^ *P* < 0.001; compared with the AD model group, ***P < 0.001.

## 4 Discussion

AD remains a major global public health challenge due to its complex pathogenesis (including Aβ deposition, Tau hyperphosphorylation, and neuroinflammation) and the limitations of single-target Western drugs. MFXD, a classic Yang-warming TCM formula, has shown clinical potential for AD, but its underlying mechanism has not been fully elucidated. This study integrated network pharmacology, molecular docking, and *in vitro* experiments to systematically explore MFXD’s anti-AD effects.

Network pharmacology may analyze the mechanisms by which drugs treat diseases from a holistic and molecular network perspective. Molecular docking uses computer simulation to predict drug–target binding and to design drugs in a targeted manner. In this study, 36 active ingredients in MFXD were screened using network pharmacology analysis, and 230 corresponding targets were identified. In the drug core target PPI network, EGFR, FOS, and MMP9 occupy pivotal positions and are also core targets involved in AD signaling pathways, indicating that these targets may be the main targets of MFXD in treating AD and play key roles in its therapeutic effects. EGFR’s aberrant activation acts as a pivotal tyrosine-kinase switch in Alzheimer’s pathogenesis, leading to Aβ deposition, tau hyperphosphorylation and neuroinflammation (Choi et al., 2023; Mansour et al., 2022). The FOS gene is differentially expressed in the entorhinal cortex and hippocampus of AD patients compared with healthy controls, and FOS protein may play a role in the development and progression of AD through interaction with proteins encoded by AD-related susceptibility genes (Chen et al., 2019). Studies have shown that MMP9 levels are elevated in the brains of patients with mid- to late-stage AD and increased MMP9 expression in the hippocampus may be involved in the development of Aβ-induced cognitive impairment and neurotoxicity (Hernandes-Alejandro et al., 2020). Abnormal accumulation of Aβ is one of the main pathological features of AD; MMP9 can impair the ability of Aβ to be transported out of the brain via the blood–brain barrier (BBB), and inhibition of MMP9 can promote Aβ elimination through the BBB (Shackleton et al., 2019). Moreover, MMP9 is closely associated with the improvement of specific neurobehavioral deficits related to AD (Ringland et al., 2021). Therefore, specific inhibitors of MMP9 may have potential in the treatment of AD.

In the “compound–target” network analysis of MFXD, quercetin corresponded to the most significant number of key targets, indicating that it is the main active component of the prescription for preventing and treating AD. Modern studies have shown that, as a plant-derived flavonoid, quercetin possesses broad anti-inflammatory, antioxidant, neuroprotective, calcium-homeostatic, and synaptic-transmission-enhancing effects and can treat neurodegenerative diseases (Ringland et al., 2021). As a naturally occurring flavonoid, luteolin possesses anti-inflammatory, antioxidant, and neuroprotective effects. Studies have confirmed that luteolin can inhibit endoplasmic-reticulum-stress-dependent neuroinflammation, thereby alleviating cognitive impairment in AD mouse models (Tao et al., 2025). Molecular docking results showed that both luteolin and quercetin can bind to the core target MMP9, with the lowest binding energy between MMP9 and luteolin, indicating the highest probability of actual binding. Moreover, MMP9 plays a causal role in Aβ-induced neurotoxicity (Mizoguchi et al., 2009). Experiments have demonstrated that matrix metalloproteinase-9 (MMP9) is involved in the processes of neuroinflammation and neuronal damage in Alzheimer’s disease (AD) by degrading the integrity of the blood-brain barrier (BBB), promoting amyloid-beta (Aβ) deposition, and exacerbating tau protein pathology. Additionally, studies have shown that the levels of MMP9 in the brain and cerebrospinal fluid (CSF) of AD patients are significantly elevated, and this elevation exhibits a positive correlation with disease severity. Consequently, MMP9 serves as a potential biomarker for disease progression and a promising therapeutic target in AD (Weekman and Wilcock, 2016; Di Lorenzo et al., 2025). To further elucidate the potential mechanisms by which these core target compounds regulate Aβ-induced cellular damage, *in vitro* experiments were conducted. The results demonstrated that various concentrations of quercetin and luteolin significantly increased PC12 cell viability. Furthermore, quercetin (20, 40, 60 μM) and luteolin (90 μM) significantly down-regulated MMP9 expression. We speculate that MFXD may exert anti-Alzheimer’s disease effects by regulating the key target MMP9 and related pathways.

GO functional enrichment analysis revealed that MFXD mainly participates in biological processes such as responses to metal ions and oxidative stress, steroid hormone and reactive oxygen species metabolism, thereby affecting neuronal structure and function (e.g., associations with basal plasma membrane and postsynaptic membrane components) through neurotransmitter release, receptor activation, and signal transduction processes to exert therapeutic effects against AD. KEGG enrichment pathways included calcium signaling pathway, TNF, PI3K-Akt, HIF-1, neuroactive ligand-receptor interaction, cAMP, MAPK, apoptosis, and NF-κB signaling pathways, among which the calcium signaling pathway is closely related to AD mechanisms and may be an essential signaling pathway through which MFXD improves cognitive dysfunction. As a strictly controlled second messenger, calcium regulates neuronal apoptosis, energy metabolism, and other physiological activities based on interactions with proteins, which is crucial for maintaining normal neuronal function. Disruption of calcium homeostasis will enhance oxidative stress, free-radical formation, and neuronal degeneration in AD, and changes in intracellular calcium may even be considered the root cause of neuronal damage and dysfunction in AD (Calvo-Rodriguez and Bacskai, 2021). It regulates physiological activities such as energy metabolism and apoptosis through interactions with proteins and also plays a key role in learning and memory processes. In addition, the tumor necrosis factor (TNF) signaling pathway and nuclear factor-kappa B (NF-κB) signaling pathway are also crucial. Studies have demonstrated that in the pathological microenvironment of Alzheimer’s disease (AD), amyloid-beta (Aβ) deposition and abnormally phosphorylated Tau protein can induce the activation of microglia and astrocytes, which in turn secrete tumor necrosis factor-α (TNF-α). TNF-α then initiates downstream signal cascades by specifically binding to TNFR1/TNFR2 receptors on the cell membrane, activating the NF-κB pathway and promoting the nuclear translocation of its p65/p50 subunits. This nuclear translocation subsequently leads to the transcriptional upregulation of pro-inflammatory factors (such as matrix metalloproteinase-9, MMP9) and matrix metalloproteinases. These upregulated molecules further exacerbate blood-brain barrier (BBB) disruption, amplify neuroinflammation, and impair neuronal synaptic function, thereby forming a vicious cycle of “pathological stimulation-TNF signaling-NF-κB activation-MMP9 upregulation-pathology aggravation.” This cascade is involved in the core process of neurodegeneration in AD (Zhao et al., 2020; Ding et al., 2020).

*In vitro* experimental results have verified the potential of MFXD and its core components (quercetin and luteolin) in AD effects. The results showed that all three could specifically ameliorate the pathological damage of AD model cells. At the level of cell viability: both quercetin and luteolin increased cell viability in a concentration-dependent manner, while MFXD exhibited both dose-dependent and time-dependent effects. The most optimal viability recovery was observed when AD model cells were treated with MFXD at a concentration of 15% (v/v) for 24 h, and this effect was slightly superior to that of the clinical drug donepezil hydrochloride (DHCL). It can thus be inferred that the multi-component synergistic effect of MFXD confers advantages over the single mechanism of action of DHCL. Western blotting analysis revealed a significant upregulation of MMP9 protein expression in AD model cells. Quercetin inhibited MMP9 expression in a dose-dependent manner, whereas luteolin only exerted a significant effect at high doses. These findings indicate that their therapeutic effects are associated with the regulation of MMP9, which serves as a key target involved in AD pathological processes (such as extracellular matrix degradation and neuroinflammation). Meanwhile, AD model cells also exhibited activation of the NF-κB pathway, hyperphosphorylation of tau protein, and abnormal activity of GSK-3β. MFXD could simultaneously ameliorate the aforementioned pathological alterations, whereas DHCL only selectively inhibited the NF-κB pathway. Moreover, MFXD showed more prominent effects in restoring the phosphorylation of GSK-3β and the dephosphorylation of tau protein, demonstrating its advantage in multi-target regulation. In addition, ELISA results showed a significant increase in TNF-α levels in AD model cells. Both MFXD and DHCL effectively reduced TNF-α secretion, further confirming their anti-inflammatory effects. In summary, quercetin, luteolin, and MFXD all exert protective effects on AD model cells. Among them, MFXD demonstrates superior performance in improving cell viability, regulating pathological proteins, and inhibiting inflammatory factors, attributed to its characteristics of multi-component, multi-target, and multi-pathway synergistic regulation.

This study has certain limitations. First, network pharmacology screening may omit some active components with low OB/DL but potential *in vivo* activity. Second, *in vitro* experiments relied on a single cell model, which cannot fully replicate the *in vivo* AD microenvironment. Future studies can validate MFXD’s efficacy in AD animal models while investigating the synergistic interactions between its three constituent herbs, which will help translate the *in vitro* findings to *in vivo* remedies and provide stronger support for MFXD’s clinical application in AD.

## 5 Conclusion

MFXD potentially exerts anti-AD effects through a multi-component, multi-target, multi-pathway approach. Its key active components (quercetin, luteolin) may act by modulating the core target MMP9. MFXD can simultaneously regulate several pathways, such as the TNF signaling pathway, Calcium signaling pathway, NF-κB signaling pathway and target Tau protein-related pathology by restoring the phosphorylation level of GSK-3β to suppress abnormal hyperphosphorylation of Tau, thereby alleviating pathological damage in AD.

## Data Availability

The original contributions presented in the study are publicly available and the data supporting this study are included in the article and its supplementary materials. The data are available from the corresponding author upon reasonable request.

## Footnotes

1
http://www.megabionet.org/tcmid/

2
https://old.tcmsp-e.com/tcmsp.php

3
http://swisstargetprediction.ch

4
https://www.uniprot.org/

5
https://david.ncifcrf.gov

6
https://string-db.org

7
https://pubchem.ncbi.nlm.nih.gov/

8
https://www.uniprot.org/

9
http://www.rcsb.org/
